# BEAT: Bioinformatics Exon Array Tool to store, analyze and visualize Affymetrix GeneChip Human Exon Array data from disease experiments

**DOI:** 10.1186/1471-2105-13-S4-S21

**Published:** 2012-03-28

**Authors:** Arianna Consiglio, Massimo Carella, Giorgio De Caro, Gianfranco Delle Foglie, Candida Giovannelli, Giorgio Grillo, Massimo Ianigro, Flavio Licciulli, Orazio Palumbo, Ada Piepoli, Elena Ranieri, Sabino Liuni

**Affiliations:** 1 Institute for Biomedical Technologies of Bari - ITB, National Research Council, Bari, 70126, Italy; 2 Medical Genetics Unit, Casa Sollievo della Sofferenza IRCCS, San Giovanni Rotondo Foggia, 71013, Italy; 3 Institute of Intelligent Systems for Automation - ISSIA, National Research Council, Bari, 70126, Italy; 4 Department and Laboratory of Gastroenterology Unit, Casa Sollievo della Sofferenza IRCCS, San Giovanni Rotondo Foggia, 71013, Italy; 5 Department of Biomedical Science, University of Foggia, Foggia, 71122, Italy

## Abstract

**Background:**

It is known from recent studies that more than 90% of human multi-exon genes are subject to Alternative Splicing (AS), a key molecular mechanism in which multiple transcripts may be generated from a single gene. It is widely recognized that a breakdown in AS mechanisms plays an important role in cellular differentiation and pathologies. Polymerase Chain Reactions, microarrays and sequencing technologies have been applied to the study of transcript diversity arising from alternative expression. Last generation Affymetrix GeneChip Human Exon 1.0 ST Arrays offer a more detailed view of the gene expression profile providing information on the AS patterns. The exon array technology, with more than five million data points, can detect approximately one million exons, and it allows performing analyses at both gene and exon level. In this paper we describe BEAT, an integrated user-friendly bioinformatics framework to store, analyze and visualize exon arrays datasets. It combines a data warehouse approach with some rigorous statistical methods for assessing the AS of genes involved in diseases. Meta statistics are proposed as a novel approach to explore the analysis results. BEAT is available at http://beat.ba.itb.cnr.it.

**Results:**

BEAT is a web tool which allows uploading and analyzing exon array datasets using standard statistical methods and an easy-to-use graphical web front-end. BEAT has been tested on a dataset with 173 samples and tuned using new datasets of exon array experiments from 28 colorectal cancer and 26 renal cell cancer samples produced at the Medical Genetics Unit of IRCCS Casa Sollievo della Sofferenza.

To highlight all possible AS events, alternative names, accession Ids, Gene Ontology terms and biochemical pathways annotations are integrated with exon and gene level expression plots. The user can customize the results choosing custom thresholds for the statistical parameters and exploiting the available clinical data of the samples for a multivariate AS analysis.

**Conclusions:**

Despite exon array chips being widely used for transcriptomics studies, there is a lack of analysis tools offering advanced statistical features and requiring no programming knowledge. BEAT provides a user-friendly platform for a comprehensive study of AS events in human diseases, displaying the analysis results with easily interpretable and interactive tables and graphics.

## Background

In biological complexity generation, the AS mechanism is a major contributor to the diversity of proteome [1,2]. Although it has long been presumed that only 5% of human genes was alternatively spliced, more recent estimates - based on experimental evidence and computational approaches using ESTs mapped onto mRNA sequences - showed a much higher rate of the phenomenon in human genes: the actual percentage of genes that exhibit AS events has grown up to 95% [3-8]. The AS mechanism is usually categorized into five basic modes: exon skipping of cassette exons, mutually exclusive exons, alternative donor site, alternative acceptor site, and intron retention. Exon skipping of cassette exons is the most common mode in mammalian pre-mRNAs, and it occurs when an exon is spliced out of the primary transcript or retained. In some cases, multiple cassette exons are mutually exclusive, producing mRNA that always includes only one of several exon choices. Defects in the AS mechanism have been involved in many diseases [9-11]. Exon array technology is a new type of microarray offering a more fine-grained chip to support global inference about gene expression at the level of individual isoforms and exons. It allows a more comprehensive analysis of the transcriptome, as well as the study of Alternative Splicing. One of the first uses of the Affymetrix GeneChip Human Exon 1.0 ST array [12] was the study of the aberrant splice variants involved in the initiation and/or progression of glial brain tumor [13]. Numerous studies followed, including amyotrophic lateral sclerosis and multifocal motor neuropathy [14], cystic fibrosis and several human cancers [15,16].

Exon arrays are one of the first available chips to survey both gene expression and AS patterns on the whole-genome scale on a single array. One exon array is a chip containing about 5.4 million probes grouped in 1.4 million probesets, each one designed to map at most a single exon. Probesets are grouped into transcript clusters that are portions of the genome roughly corresponding to genes.

The output of an Affymetrix Exon Array is a binary CEL file containing probe level intensities from a single array. Affymetrix offers a toolbox essential for CEL files analysis, the Affymetrix Power Tools (APT) [17], and a set of library files with information useful for the preprocessing of raw data and the annotation of probesets and transcript clusters. Using APT, we extract numerical expression intensities for each probeset (called "exon level" expression), a summarization of transcript cluster expression intensity computed from its probesets ("gene level" expression), a quality assessment of each probeset value (DABG: Detection Above BackGround [18]) and a statistical value for the AS detection (MiDAS: Microarray Detection of Alternative Splicing [19]). For the analysis of AS events in the comparison between normal and pathological tissues, Affymetrix suggests as a standard analysis his MiDAS and classical t-test statistics. The evaluation of the AS trend is computed by the Splicing Index, a ratio between normal and pathological exon expression, each of them normalized on the overall gene level expression.

Other statistical algorithms, such as MADS [20] and FIRMA [21], have also been proposed. These methods focus their analysis on statistical computations, providing the users with command-line applications and requiring prior knowledge of statistical programming languages like R. AltAnalyze [22], easyExon [23] and Exon Array Analyzer [24] are the most recent tools for exon array analysis. AltAnalyze, when installed with DomainGraph plug-in of CytoScape, is a complex software workflow for the statistical and visual analysis of exon expression data, and it requires a minimum of 1 GB of RAM and from 1 up to 3 GB free hard-drive space for species gene databases, Affymetrix libraries and annotation files. Another stand-alone software is the java-based easyExon, which offers fewer facilities, as it provides expression statistics results with only a few biological annotations such as gene and GO annotations for probesets. Exon Array Analyzer is a web tool that allows the user to upload his CEL files and shows tabular exon and gene level expression results, together with MiDAS and FIRMA output.

The heaviest drawback of stand-alone software packages for exon array analysis is the huge requirement in RAM and hard disk space. They ensure privacy of data but they need an expensive setup and advanced programming skills for a flexible analysis. The available web tools, for their part, are not very complete as they lack in advanced analysis instruments. The most important limit of all the existing tools providing a graphical interface is forcing the user to input also some analysis parameters such as p-value thresholds and AS extraction algorithm option during the upload of CEL files. It means that the user must choose his analysis parameters even when he does not know how they will influence the results, and even if he wants to change just one parameter, he must restart the entire analysis process.

The aim of BEAT, the platform we describe in this paper, is to provide the scientific community with a user-friendly platform to analyze exon array datasets with rigorous statistical methods and an easy-to-use graphical user front-end. BEAT has been developed as a web tool because we think that the Internet is the most important means for spreading research results, using only a browser and the Internet connection (today even on mobile phones). At the same time we put attention on the security and privacy of data and result transfer. BEAT simplifies the exon array analysis workflow asking no preliminary parameters and displaying the results by interactive plots and tables. Furthermore, it introduces some new instruments to obtain very useful and easily interpretable results for each case study, such as the novel use of meta statistics and the possibility to exploit other clinical information about the patients for a multivariate analysis of exon expression intensities.

## Implementation

### Architecture of the platform

A top-level view of BEAT has the same structure of a classical web application, since the platform was designed according to the typical three tier architecture. This approach allows a modular, scalable, extensible, and easily administrable system architecture, in order to guarantee the interoperability among the components.

1. **Data**. The first tier consists of a data warehouse. The data warehouse stores all data sources and statistical processed data allowing smart data storing and efficient data retrieving. It consists of more than one relational database, a repository/staging area and data marts. It is described in detail in the "Data Warehouse" section and in the Additional file 1.

2. **Service-integration**. The second tier consists of an application server (Tomcat) dedicated to the deployment of the web interface and a set of applications. Such applications perform both exon and gene level analysis through the APT and the statistics and meta statistics using complex R routines, as described in the "Analysis workflow" section.

3. **Web front-end**. The third tier consists of the web query interface, which is developed for displaying the analysis results and browsing the data contained in the Data Warehouse, in order to provide interactive plots and a flexible and advanced query system.

**Figure 1:**
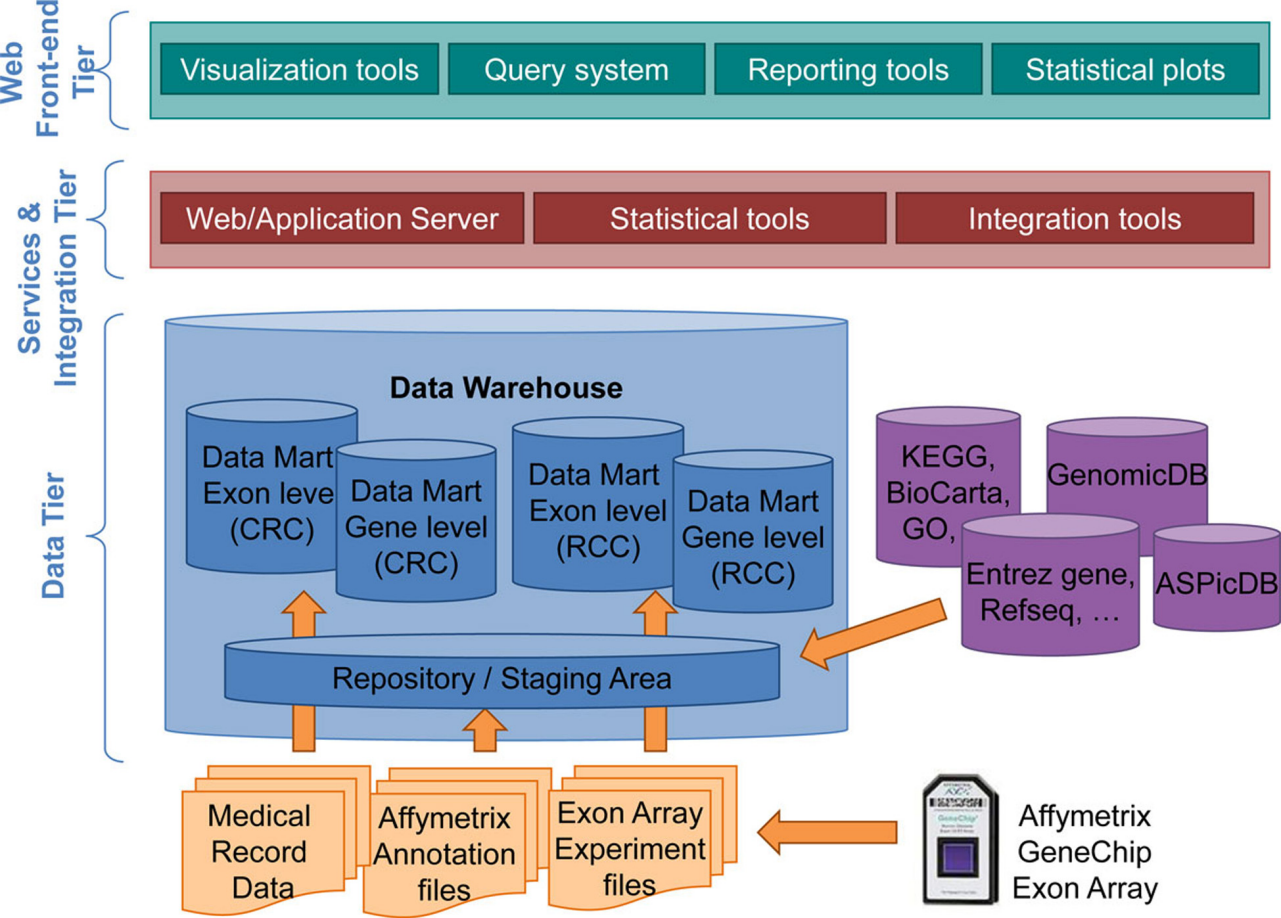
**Architecture of the BEAT platform**. BEAT was designed using a typical three tier architecture. The first tier consists of a data warehouse, which stores all data sources and statistical processed data allowing smart data storing and efficient data retrieving. The Service-integration tier consists of a Tomcat application server and a set of applications performing exon and gene level analysis. The web front-end tier is the query interface, with its advanced query system and interactive plots.

Figure 1 represents the graphical schema for the architecture of BEAT.

From the user perspective, the platform is designed to analyze a user "case study" in a workflow that starts from a set of Affymetrix exon array experiment output files (CEL files) and ends with the visualization of the statistical analyses of gene differential expressions and exon splice variants.

### Data sources

The data sources used by BEAT are stored as relational tables in the data-warehouse component and they can be classified in different types:

- **User data file: **the outputs of Affymetrix Exon Array experiments (CEL files), which are binary files containing probe-level intensities from a single array; a text file filled by the user through a web wizard containing metadata for each CEL file, including medical record information about a patient, such as gender, age of disease onset, tumor type and tissue, etc. These files are interpreted and elaborated by Affymetrix APT tools.

- **Affymetrix annotation files: **text files contain both design-time information and NetAffx [25] mapping between probesets and public mRNA sequences (cDNAs). These annotations include statistical information specific to the probeset composition and sequence annotations at both exon and transcript level extracted from public databases. In the platform we have used the Relese29 - hg18 version.

- **Public database: **different public biological databases stored in the data warehouse come from structured and unstructured sources like external database dump or text/CSV files. They are: HUGO Gene Nomenclature Committee (HGNC) database [26] reporting information about official human gene names and aliases; KEGG [27], BioCyc [28] and BioCarta Pathways [29] for the association among genes and biological pathways involved; Gene Ontology (GO) database [30], that provides a controlled vocabulary of terms which describe gene product characteristics and gene product annotation data.

- **Specialized database: **ASPicDB [31], a database designed to provide information and reliable annotations of the AS pattern of human genes; FeatDB, a custom database reporting chromosomal location about known (RefSeq) human transcripts extracted from UCSC genome browser [32].

### Analysis workflow

**Figure 2:**
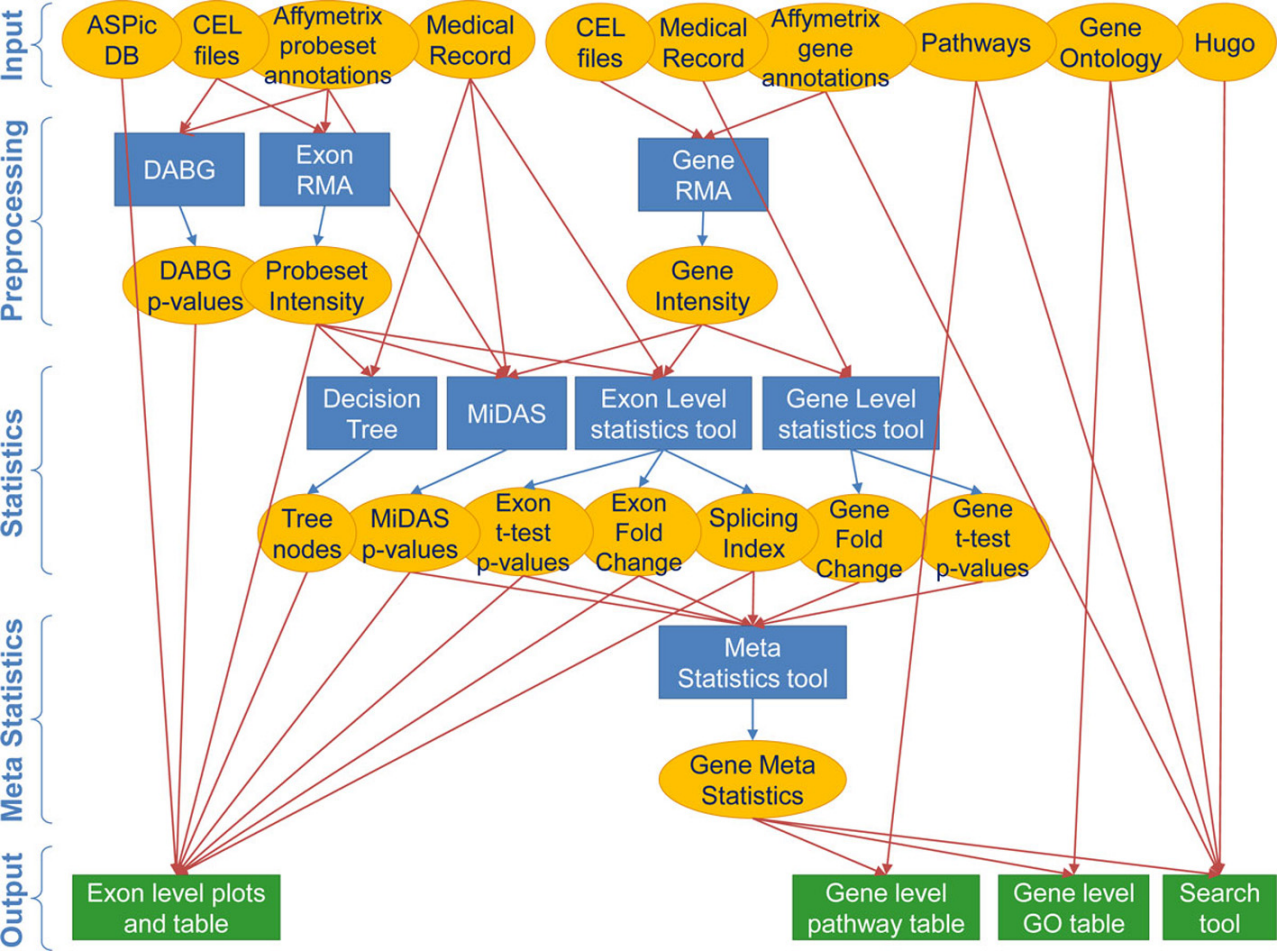
**BEAT analysis workflow**. Elliptical boxes represent every type of data sources, blue rectangles represent processes and applications, with differently colored arrows for input and output flow. Green boxes represent front-end result visualization. For graphical reason only, CEL files and Medical Record input are duplicated for Exon and Gene processing.

Figure 2 shows the analysis workflow for the entire bioinformatics process performed on Affymetrix Exon Array datasets. The first steps involve CEL file preprocessing using APT. Starting from raw binary CEL file we extract probeset and transcript cluster expression intensities performing a Robust Multi-chip Analysis (RMA) summarization. All the other statistical computations are performed using R [33].

Each probeset is designed to map only one exon or a part of it and it can be used for the exon level analysis. The transcript cluster represents groups of transcripts falling in the same portion of a chromosome. These summarization expression values are used for the exon level analysis, because values of the same probeset coming from different CEL files can be compared normalizing them on their transcript cluster expression.

The transcript cluster values are not very accurate metrics for gene level analysis, because they often group together different genes sharing only a few probesets, assigning them the same expression value. For this reason, we have slightly modified RMA parameters in order to obtain a more correct gene expression profile, computing the expression of each gene using only the probesets mapped to its known isoforms.

Unlike the other existing Exon Array tools, in our workflow the exon level and the gene level results merge into the use of meta statistics that are introduced to evaluate the results and to explore the data. The following paragraphs describe all these steps in detail.

#### Exon level analysis

In exon level analysis, the normalized probeset expression intensities are used to study changes in exon expression when comparing two or more experimental groups in order to find out the AS events correlated to the groups.

The most common studies compare normal to pathological tissues. For this type of analysis in which only one variable is involved, we perform all the standard statistics on each probeset: the Splicing Index (logarithmic ratio between normal and pathological normalized exon expression), useful to evaluate the trend of the expression changes; the Student's t-test; the MiDAS, the Affymetrix algorithm to extract the p-value. We also compute the Fold Change on probeset intensities not normalized for the overall gene expression level.

For a deeper analysis of the AS events, we provide alternative isoforms of the gene under investigation, for both known and predicted alternative transcripts.

In order to perform a quality estimation of each statistical result obtained at exon level, we allow the user to filter data according to the Affymetrix DABG p-value estimation.

#### Multivariate AS analysis

Another interesting study in Alternative Splicing is multivariate analysis, in which AS events are evaluated in relation to more than one clinical variable, such as gender, stage of the pathology or age of disease onset. Affymetrix proposes the use of ANOVA [34], which is widely used by biologists and clinicians in several experiments as prognostic significance of tumor states. In exon array experiments the multivariate analysis is often set aside, mainly because of the lack of data on CEL files variables other than normal-pathological information and the complexity of repeating the ANOVA test and its p-value correction on thousands of probesets at the same time.

In our platform, we have included a component for multivariate analysis that simplifies the application of ANOVA, providing an easily interpretable output of the multivariate analysis. The methodology is based on a well-known data mining paradigm: the Decision Tree Algorithm [35,36]. In order to manage both numerical and symbolic data, we use a conditional inference decision tree [37], training it on normalized expression intensities. Conditional inference trees estimate a regression relationship by binary recursive partitioning in a conditional inference framework. Roughly speaking, the algorithm works recursively repeating the following steps: it tests the global null hypothesis of independence among any of the input variables and the response; it stops if this hypothesis cannot be rejected, otherwise it selects the input variable with the strongest association to the response, measured by a p-value corresponding to a test for the partial null hypothesis of a single input variable and the response; it implements a split in the selected input variable. The implementation uses a unified framework for conditional inference, or permutation tests [38].

The output of the algorithm is a tree graph in which each node is a variable that influences the changes in expression intensities. If the variable is binary, the node splits the data according to its two values, while if the variable is numerical, the node indicates a threshold correlated to a significant change in expression intensity. The rules for the generation of nodes and for the pruning of the tree are based on ANOVA.

For example, if expression signal of a probeset reveals a correlation both to Male/Female comparison and to a threshold of 60 as age of disease onset value, the tree highlights in its output the two variables and the threshold, suggesting to the user AS events correlated to non pathological characteristics.

#### Gene level analysis

Gene expression intensities are summarization values computed from probeset intensities. In order to evaluate the changes in gene expression profile, we compute the Fold Change ratio to compare normal to pathological issues, validated by means of the t-test p-value.

The gene level value is not a very informative index in the AS events discovery, as it characterizes the whole gene differential expression. We introduce the use of meta statistics to overcome this drawback and to obtain a method to compare genes having different characteristics.

#### Meta statistics

Meta statistics are descriptive metrics used to provide interpretable information describing the expression profile of all probesets belonging to one gene. For example, if a gene is subjected to an AS event, we will see a very low p-value for the probesets involved in the AS, and higher values for the unaltered probesets. In terms of meta statistics, this can be represented with a minimum p-value tending to 0 and a mean p-value tending to 1.

Therefore, the meta statistics are defined as minimum, maximum, mean, and variance values, which are computed on the standard exon level statistics results. The default values of the search for AS events are given into the search page of BEAT. Together with gene level results, the meta statistics have been used with a sorting algorithm in order to cluster together genes according to their characteristics.

This method has been borrowed from the application of clustering algorithm as used in many bioinformatics approaches. When we have to process a huge number of data, it is often useful to group the ones with similar characteristics into clusters. Similarity is evaluated by means of a distance metric. Our idea was to exploit such a distance metric to extract genes with specific characteristics, i.e. choosing the center of the cluster and analyzing the nearest genes. Meta statistics have been designed to be used for this type of distance comparison, and the distance metrics used is the Euclidean distance with variables scaling, because it allows a very quick distance computation for thousands of multi-dimensional points [39].

Meta statistics and the sorting by Euclidean distance have been applied in the BEAT platform both to optimize the search tools provided for result exploration and to analyze the expression profile of genes belonging to the same pathway or mapped to the same Gene Ontology term.

### The Data Warehouse

The data management in BEAT is delegated to a data warehouse (DW). A DW is defined as "a subject-oriented, integrated, non-volatile and time-variant collection of data in support of management's decisions" [40]. The data in the warehouse are filtered, aggregated and stored in smaller data storages, usually called data marts (DM), properly designed for specialised purposes. A DW is frequently used in business applications but in the last years it is often used also in the biomedical (especially clinical) domain [41-44]. The choice of a DW for BEAT data management was driven by the following aspects:

- The DW is a consolidated database technique, suitable for storing the large quantity of experimental data produced by exon array experiments. A single case study produces 1.4 million probeset signals for each chip (stored as records in a database table) and the same number of results for each statistical analysis performed on these signals.

- The DW architecture facilitates integration of locally produced experimental data with public bioinformatics databases used as functional annotation extensions (the biological background knowledge), with the aim of easily producing new knowledge.

- A DW allows multidimensional On Line Analytical Processing (OLAP) techniques to support data mining, statistical analyses and reporting functionalities that are normally not feasible with typical transactional databases approaches (OLTP). The OLAP functionality adapts well to the complex analytical procedures implemented in this tool.

BEAT DW complies a three-tier architecture. The statistical analysis design implemented in BEAT has led to the definition of two data marts (**BEAT_exp_exonlevel **and **BEAT_exp_genelevel**) that support the analytical processes of the exon and gene level analyses described in the previous section. In addition, a repository was implemented (named **BEAT_Repository**), where the input data sources (see "Data Sources" paragraph) are stored, processed, homogenized, and reconciled in order to facilitate the data mart population.

The physical tables belonging to the data marts and the repository have been populated through the use of an Extract/Transformation/Load (ETL) tool usually used for this purpose in DW systems.

For the development of the DW we used MySQL Rel. 5.× and Infobright [45] ICE 4.0 (a column-oriented relational database engine integrated with MySQL dedicated to DW system) Relational Database Management Systems (RDBMS), while to implement the ETL process we used the open source tool Pentaho Data Integration (aka Kettle [46]), a component of the Pentaho Open Source Business Intelligence.

The data marts were designed using the fact constellation schema conceptual model and adopting the standard Dimensional Fact Model graphical annotation [47].

Supplementary information about the repository and the data marts can be found in Additional file 1.

### System deployment process

**Figure 3:**
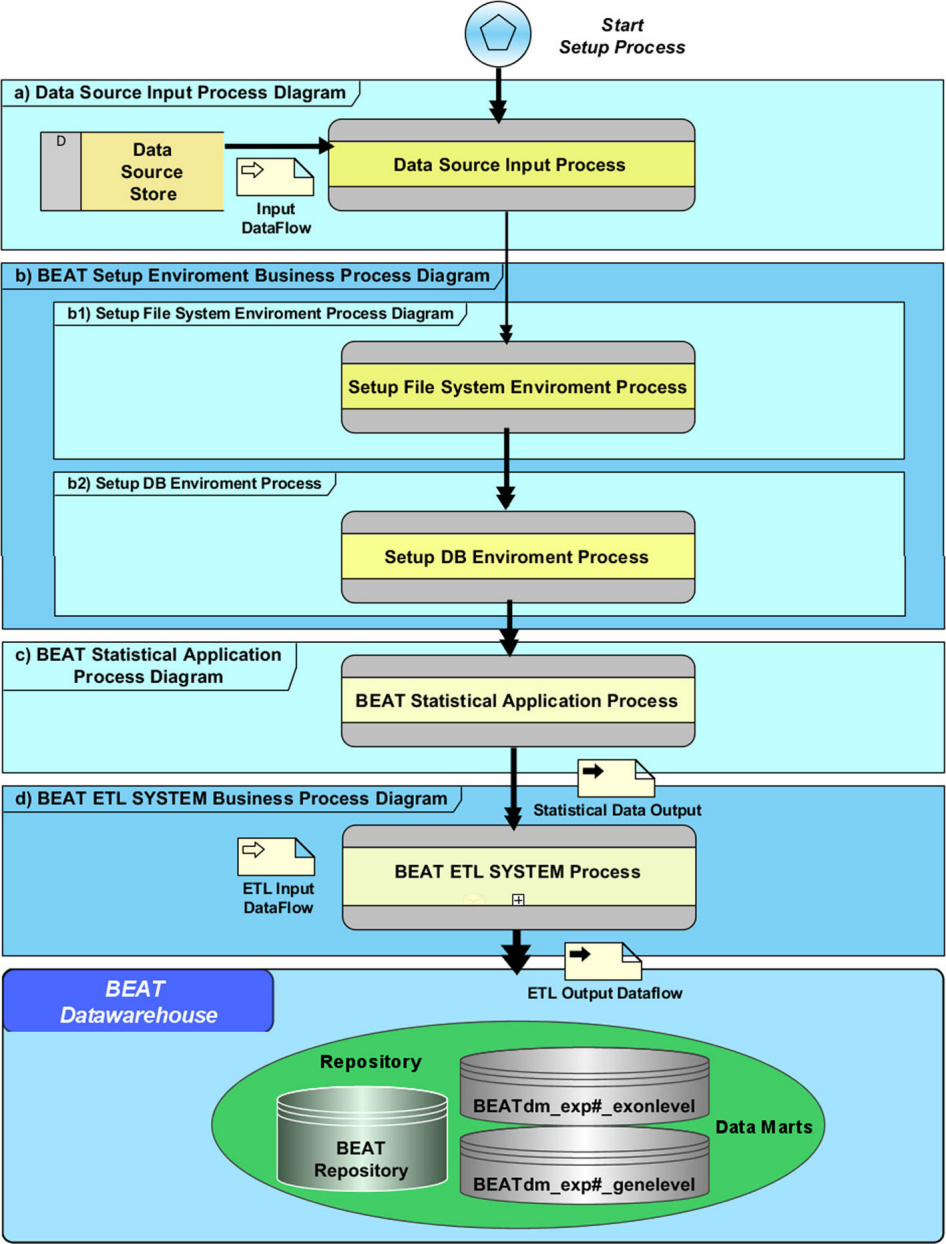
**BEAT deploy system lifecycle business process diagram**. UML diagram of BEAT system deploy process. Each box represents a process, eventually composed by sub-processes, implemented by the Kettle ETL tool. The diagram emphasizes the logical sequence of processes where the arrows represent the process flow. In the diagram, the main data flows involved in the system deploy process are also represented.

In BEAT, the analysis of a user case study corresponds to an execution of a pipeline process to deploy all data transformation and statistical analyses performed by BEAT components starting from the users' experimental data (exon array CEL files). The entire process of setup, initialization, deployment and commissioning of a case study is described by the BEAT Deploy System Lifecycle Business Process Diagram shown in Figure 3. It is structured in a hierarchical way where each block can be blown-up in sub-diagrams. The process diagram is composed by the following macro steps:

1. **Data source input process**. It is the process delegated to extract all the data sources needed by a case study: CEL files, medical record file, Affymetrix Exon array annotation files and all the public and specialized database listed in the "Data Sources" paragraph. The files and database extracted are used by the ETL process to populate the repository and data marts (Figure 3-a).

2. **Setup file system environment process**. This process initializes all the environment variables and creates the directory tree that will contain all input and processed case study files (Figure 3-b1).

3. **Setup DB environment process**. This process creates the new two empty BEATdm_exp#_exonlevel and BEATdm_exp#_genelevel data marts related to a particular case study identified by the "#" id in the CaseStudy metadata table (Figure 3-b2).

4. **BEAT statistical application process**. It manages the statistics and meta statistics process analyses for gene and exon level. It is composed by two sub-processes: APT Process and R Process (see Figure 2). The process starting from the input dataflow (CEL files and medical records) produces the statistical analysis files (Statistical DataOutput) that will populate the fact tables of the data marts (Figure 3-c).

5. **BEAT ETL system process**. It contains all ETL sub-processes that, starting from the input dataflow (statistical analysis output files, medical record file and data sources extracted by the first step), populate the tables of the BEAT repository, BEAT_exp#_exonlevel and BEAT_exp#_genelevel data marts (Figure 3-d).

The processes described in the BEAT Deploy System Lifecycle Business Process Diagram have been implemented by means of software components, named "job" and "transformation", using Kettle (described in "The Data Warehouse" section). The whole process of input data extraction, statistical analysis and data warehouse population, is run by a single Kettle master job launched by the BEAT system, after CEL files uploading. The master job, where the right sequence of ETL components is highlighted, is graphically presented in Figure 3.

### The web front-end

BEAT provides an easy-to-use interface for the Affymetrix Exon Array datasets submission, a storage and retrieval system, and interpretable outputs in terms of figures and tabular data, using a web browser and the Internet connection.

The platform has been developed using the Zkoss framework [48], which is a client-server Java-based technology. Zkoss shields from the complexities in classical Ajax/Javascript approaches, focusing the developer on the application logic, and delivering the user interfaces within standard web browsers.

It runs on Apache Tomcat at the server side as a cross-browser client engine responsible for the rendering of the front-end, which interacts with the application server and handles events, communication and AJAX duties. The client interface is also compatible with various mobile browsers.

The user interfaces are defined using a XML markup language, and their functionalities can be extended with embedded java code and/or integrated with many popular frameworks such as Spring, JasperRepors, Hibernate and so on.

#### CEL files uploading

If the user wants to upload exon array files, he needs to be registered with a valid e-mail address. Once logged in, he can start the upload procedure that guides the user in sending CEL files to the system. Clinical data can be associated to each CEL file by filling a form: the user must specify at least if the CEL file comes from normal or pathological tissue and, if available, he can add information about gender, age of onset of the pathology, stage of the disease.

Once successfully loaded all the data, the user can set the start of the analysis. CEL files and clinical data will be preprocessed and analyzed, and the analysis results will be loaded into the data warehouse. Once the process is completed, the user will be notified by e-mail.

Each user is allowed to see only the results of his own provided CEL files, unless these files have been marked for public release during the upload phase.

In order to ensure the compliance with the national laws and decisions from the Italian Data Protection Authority, submitters are not allowed to provide any personal information (i.e. family/first name) and they can only associate a numerical id with each CEL file, if they need to create a link with their patient's clinical record.

A daily backup of submitted data is performed, in order to prevent data-loss on hardware faults. An important aspect we have implemented in our case study creation process is that the user is not asked to insert any analysis parameters, such as p-value thresholds, or to choose the AS extraction algorithm. In fact, the tool performs all the standard analyses on the data and provides all the results, showed using interactive plots and summary tables. All private case studies will be deleted if they have not been accessed for 18 months.

Once the automated analysis process performed on the case study is completed, the user can explore the results starting from the search page. The result visualization, in fact, is organized in just two steps: in the first step we offer an advanced search tool to provide the user with an intuitive and comprehensive way to search through the data and to choose a list of interesting genes; in the second step, the user can visualize all the results of the analysis performed on a gene, at both exon and gene level.

#### The search page

**Figure 4:**
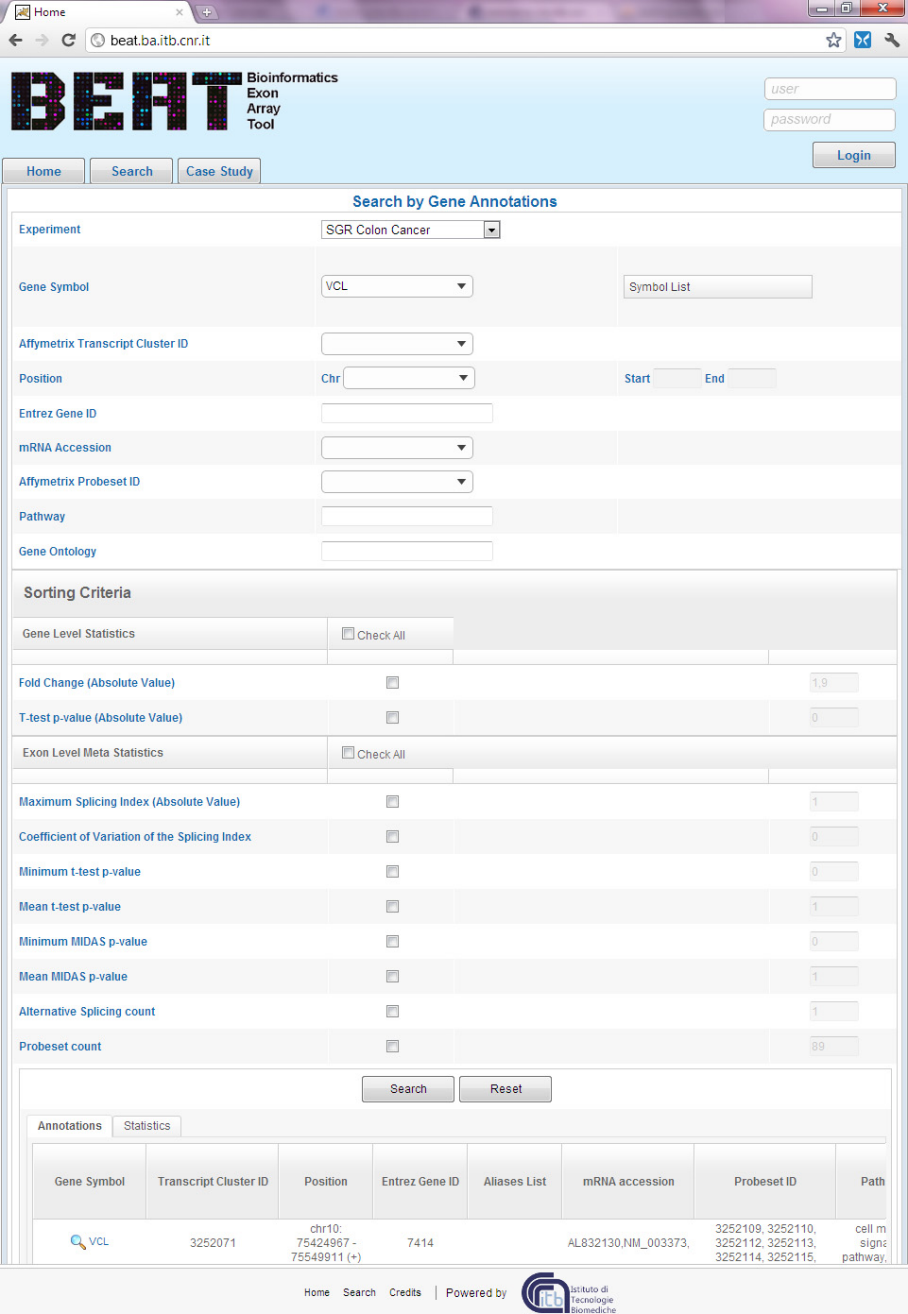
**Gene search page**. This screenshot of the web front-end shows the page that allows the search through the data. Gene annotations can be used to retrieve a list of examined genes (appearing as a table in the bottom of the page), while the meta statistics can be exploited to order the rows of the result table.

BEAT search page is a comprehensive instrument for exploring the results of the analysis carried out on each case study. As Figure 4 shows, it offers two main instruments useful in the AS events mining: a search form for retrieving genes with selected properties, and a set of sliders for meta statistics values, provided to order data by a selected statistical behavior. The activation of meta statistics sorts the results according to the previously described Euclidean distance. The exon level meta statistics, initialized with default values, help to find out interesting AS events: the user has just to check all the exon level meta statistics and start the search, to obtain a list of genes with potentially interesting splicing events, sorted by relevance.

For example, if the user wants to investigate the results of a particular gene, he can insert in the annotation form one or more information useful to retrieve it or if he wants to analyze all the gene with differential expression belonging to a particular pathway, he can select the pathway and order the data activating meta statistics by inserting a high Fold Change Value and a low t-test p-value. To search for isolated AS splicing events in all genes belonging to chromosome 8, the user can select the chromosome leaving start and stop position blank, and exploit meta statistics to bring out data with a low p-value for t-test and MiDAS and only one or two probesets revealing AS events. The search results are visualized in a table with one gene by line satisfying the search criteria, on the bottom of the page. Each row of the table is linked to a page containing the detailed results of the analyses carried out on the selected gene.

#### Gene result page

**Figure 5:**
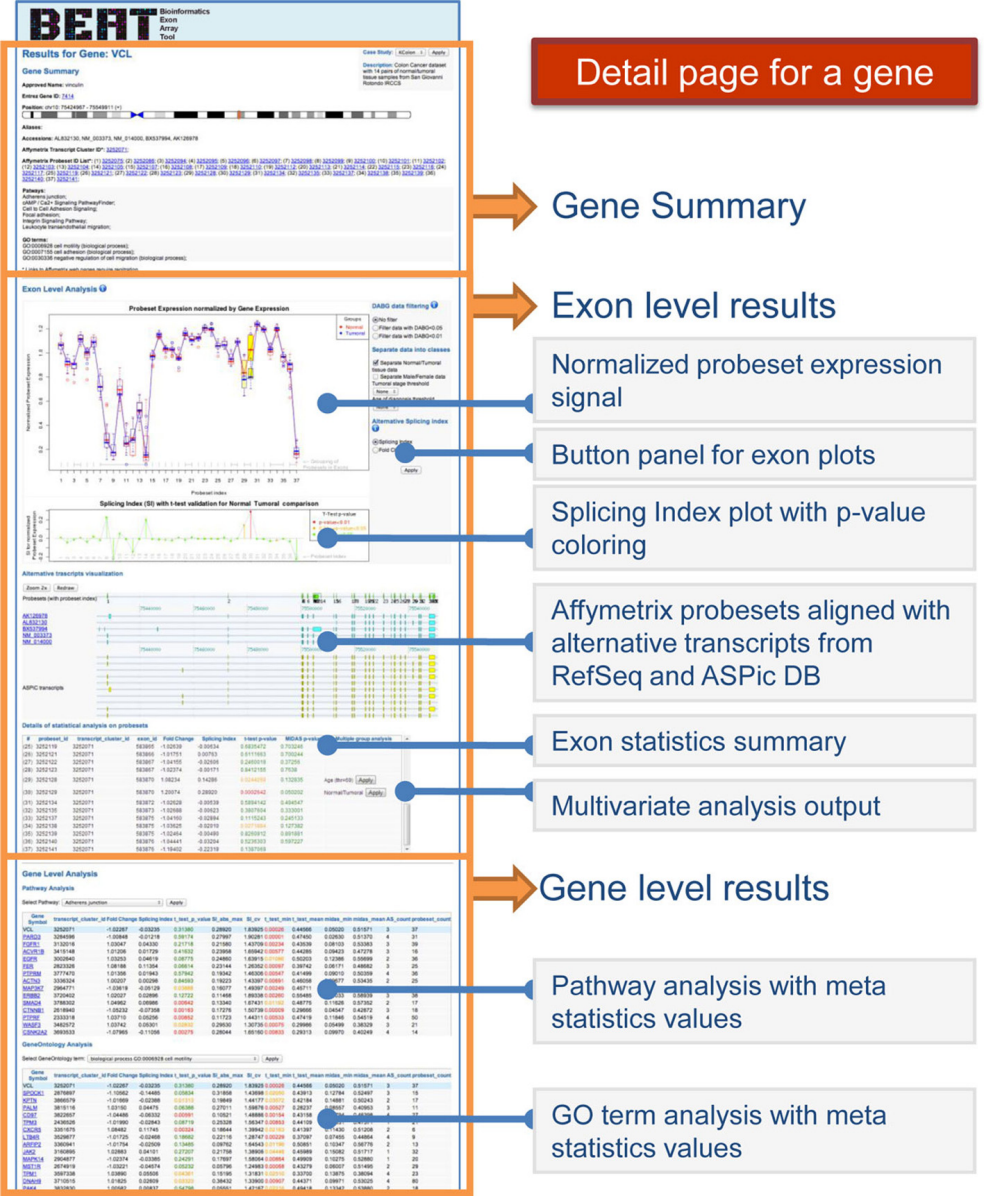
**Gene result page**. The detail page for the analyses performed on a gene and its exons is composed by three sections. These sections are highlighted with orange boxes and their content is detailed by the grey labels.

Each gene result page is composed by three sections, as shown in Figure 5. On the top of the page we have a summary of the information of the gene, such as name, position on chromosome, Affymetrix identifications with links to Affymetrix website, a list of pathways in which the gene is involved and the Gene Ontology terms.

In the second section we show the exon level analysis results and statistics about probeset expression intensities. For probeset normalized expression comparison among experimental groups, we have chosen boxplot representations, because they offer an intuitive visualization of the distribution of data with identification of outliers. The probesets that show a statistically significant discrepancy in expression intensity are highlighted in yellow. The second plot shows the trend of the Splicing Index or Fold Change, showing positive peaks where the normal data signal is higher than the pathological one, and negative peaks for the converse. AS index values are drawn with a traffic light coloring that indicates the p-value support of the data separation.

These plots are interactive and can be managed using the button panel on the left. For example, it allows applying DABG filter on the data used in the plots. We can also choose experimental groups exploiting medical record variables, in order to visualize boxplot separation into user defined classes. To make a two class comparison (single variable analysis) we can choose the proper AS index in the second plot. The parameters for the classes (age, gender, stage) are not fixed: they are automatically generated using supplementary information entered during CEL file uploading.

In the second section we also report, aligned to their portion of chromosome, the representation of probesets, RefSeq isoforms and ASPiC predicted isoforms. These images are useful for an immediate interpretation of AS events, highlighted in the previous plots, and their possible influence on different isoforms.

A summary of all the evaluation carried out on each probeset is given in a table. In the last column, we propose statistically significant class separations computed by the conditional inference decision tree, with a button that updates probeset expression plots applying the suggested experimental groups.

In the last section of the gene page we have the results of the gene-level analysis and the values of the meta statistics computed for the gene and for a cluster containing genes belonging to the same pathway or gene ontology.

Using a drop-down menu, the user can select a pathway in which the gene is involved. The system shows the other genes belonging to the same pathway, sorted by Euclidean distance, so the genes that (statistically) behave similarly to the gene under examination are listed in the first rows.

We have the same table also for Gene Ontology terms. Each gene name is a web link that opens its detail page in a new window, to facilitate the comparison with the first gene examined.

## Results

BEAT has been tested on two new datasets of exon array experiments coming from colorectal cancer and renal cell cancer experiments, produced at Medical Genetics Unit of IRCCS Casa Sollievo della Sofferenza. The Colorectal cancer dataset is composed by pairs of normal and tumor colon specimens from 14 colorectal cancer (CRC) patients undergoing curative resection at the IRCCS Casa Sollievo della Sofferenza. None of the patients suffered from hereditary CRC or had received preoperative chemo-radiotherapy. The renal cell cancer dataset is composed by pairs of normal and tumor renal specimens from 13 renal cell carcinoma (RCC) patients.

All patients gave their informed consent to take part in this study. The study was approved by the Hospital Ethics Committee.

Both the datasets were profiled by the Affymetrix Human Exon 1.0 ST Array (5.4 mln probes; 1.4 mln probesets) and anonymous information about gender, age and cancer grading were collected from the medical records of the patients.

In order to test the performances of the platform, we have also uploaded a third case study containing 173 CEL files from colorectal cancer samples. This is a public dataset and it has been downloaded from ArrayExpress (E-GEOD-24551).

The three case studies have been imported in BEAT and their analysis results are publicly accessible and allow the user to explore all the features of the platform.

**Figure 6:**
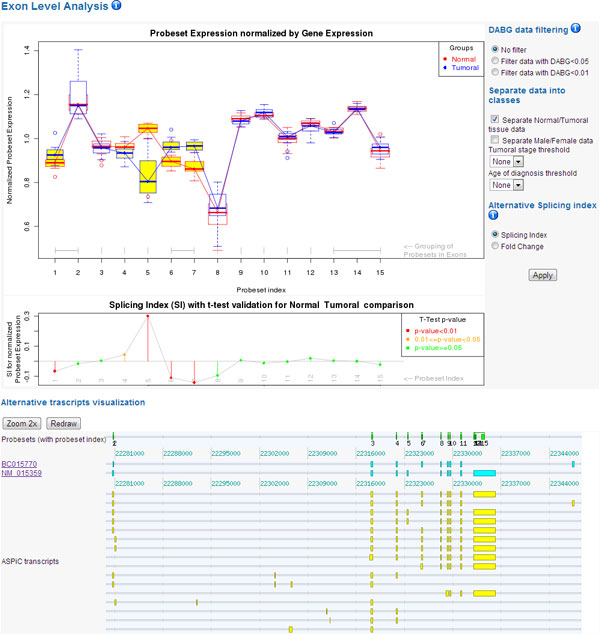
***SLC39A14 *example**. This screenshot is the "exon level" section of the result page for *SLC39A14 *gene. Probeset boxplots highlighted in yellow are aligned to red peaks in the Splicing Index. Alternative transcript representation displays that exons mapped to probesets 5 and 6-7 are mutually exclusive probesets.

Figure 6 shows the results on a gene known to be correlated to CRC, the solute carrier family 39 (zinc transporter) member 14, *SLC39A14 *[49]. Probeset expression plots reveal some evident AS events. In particular, we can see an over-expression of probeset 5 (corresponding to the fourth exon) and an under-expression of probesets 6 and 7 (the fifth exon). This phenomenon represents the well-established case of mutually exclusive exons for CRC tissues, and it is supported by opposite peaks in Splicing Index and small p-values results. Moreover, the AS event is also supported by the alternative transcripts in which the fourth and fifth exon never co-occur.

Using the dropdown menu on the top right of the page, the user can easily switch between all his "public" or "private" case studies to monitor the different behavior of a selected gene.

## Discussion

Since the very first requirement analysis designed with biologists and clinicians, it emerged that the main features of the tool would have been ease of use and rapid access to interpretable statistical analysis results.

We have kept in mind these requirements developing a web application (paying attention to private data management) in which the user could perform each process of his study through few steps. In the case study loading procedure, for example, the user has only to upload his CEL file and the available clinical information, while all the other existent exon array tools ask for some analysis parameters immediately after CEL file selection. For instance, during the CEL file uploading in Exon Array Analyzer (another existing web tool for exon arrays), the user must define three sets of initial parameters: at first he has to map each CEL to non intersecting groups, then he has to define comparisons between coupled groups, and at least he must choose some threshold for the analysis algorithms. Then the analysis flow starts and the initial parameters can be changed only restarting the entire uploading procedure. Our analysis flow does not require initial parameters because it is designed to include all the statistical examinations. Threshold values can be chosen from the user when visualizing the final interactive plots, in order to see how the results change when varying the thresholds without reiterating all the analysis workflow.

A quick access to all the data is enabled by the data warehouse architecture underlying the tool. It integrates pre-calculation steps exploiting the use of data marts and fact tables. A comprehensive search page is provided to help the user retrieving the most important analysis results. All the other tools working with exon array lack in this feature; EAA, for instance, allows the user to search through the data only by gene symbol and by platform dependent identifiers defined by Affymetrix.

Finally, the architecture of BEAT has been conceived to manage scalability of data and analysis tools. Data scalability is guaranteed by the architecture of the data warehouse, in which each case study is stored in independent data marts and the system performances are not influenced by the growth in size of the data warehouse.

At the same time, the analysis workflow design allows an easy inclusion of new statistical tools that could became standard in exon array experiments.

## Conclusions

With the progress of massive production of biological data, the bioinformatics community has to deal with a growing need of easy-to-use applications for managing a huge number of data.

BEAT provides a user-friendly application for a comprehensive study of Affymetrix Exon array data about human diseases. It offers useful analysis tools requiring no programming knowledge, and it shows the results with easily interpretable and interactive tables and graphics. The analysis workflow provides rigorous statistical methods performed on exon array data, and the results are stored in a data warehouse to ensure the optimization of the data retrieval process. The introduction of meta statistics offers a novel means of exploring results through a set of metrics that summarize gene and exon level expression statistics. AS events can be studied by comparing normal to pathological tissues and by performing a multivariate analysis on available medical record information, allowing biologists and clinicians to investigate changes in splicing patterns from a wider point of view.

The architecture chosen for the development of BEAT allows the improving of the platform with additional features and with a minimum programming effort. Some future developments are: integrating new statistical methods for AS analysis (like FIRMA); improving gene level analysis, in order to allow comparisons between exon arrays and microarrays results; extending the analyses to other exon array platforms and organisms.

## Availability and requirements

BEAT is a web platform and it is freely accessible at http://beat.ba.itb.cnr.it.

The application has been tested with the latest versions of the following Internet browsers: Firefox 7, Chrome 14, Internet Explorer 9, Safari 5, Opera 11.

## List of abbreviations used

APT: Affymetrix Power Tools; AS: Alternative Splicing; BEAT: Bioinformatics Exon Array Tool; CEL: Affymetrix Exon Array output file extension; CRC: colorectal cancer; DB: Data Base; DM: Data Mart; DW: Data Warehouse; ETL: Extraction, Transformation, Loading; R: is a language and environment for statistical computing and graphics; RCC: renal cell carcinoma; RMA: Robust Multi-chip Analysis.

## Competing interests

The authors declare that they have no competing interests.

## Authors' contributions

Conceiving of the study: SL. Coordination of the work: AC, SL, FL. Software architecture design: AC, FL, GG, MI, CG. Statistical analysis integration: AC. Data Warehouse design and development: FL, GG, GDF, GDC. Web front-end design and development: AC, MI, CG, GG. Exon array laboratory experiments: MC, OP, AP, ER. Draft contribution: AC, SL, FL, GG, GDF, MI, CG, MC. All authors read and approved the final manuscript.

## Supplementary Material

Additional file 1 **PDF file containing supplementary documentation about the data warehouse**. In particular, we report a more detailed description of the repository and data marts, and four tables with a detailed description of fact tables, hierarchies and dimensional tables. Click here for file
