# Supplementary material for: BEAT: Bioinformatics Exon Array Tool to store, analyze and visualize Affymetrix GeneChip Human Exon Array data from disease experiments
Source: BMC Bioinformatics. 2012 Mar 28;13(Suppl 4):S21. doi: 10.1186/1471-2105-13-S4-S21 (PMC3314565; doi:10.1186/1471-2105-13-S4-S21)
Supplement: Additional file 1 — PDF file containing supplementary documentation about the data warehouse. In particular, we report a more detailed description of the repository and data marts, and four tables with a detailed description of fact tables, hierarchies and dimensional tables. [file 1471-2105-13-S4-S21-S1.doc]

# Additional documentation

## The Data Warehouse

The data management in BEAT is delegated to a data warehouse (DW). A DW is defined as “a subject-oriented, integrated, non-volatile and time-variant collection of data in support of management’s decisions”.

In the DW approach data are extracted from several operational databases, and then transformed, cleansed and loaded into a multidimensional database. The data in the warehouse may be further filtered, aggregated and stored in smaller data stores, usually called data marts (DM), for specialized purposes. Thus, DWs are consolidated repositories for historical data and, in order to optimize analysis and reporting applications performances, they allow the use of specific techniques such as multidimensional data modeling, which organizes the database entities into facts and dimensions. As mentioned above, the data marts can be seen as small local DWs, which replicate and eventually synthesize a portion of the primary DW for particular application fields.

BEAT DW complies a three-tier architecture. The statistical analysis design implemented in BEAT led to the definition of a repository and two data marts.

## The repository

In the DW approach, the repository collects and integrates the tables that represent the metadata supporting the processing of the basic information into the DW. The repository is structured to describe the information on data sources, transformations, constraints and user profiles. The most important tables contained within BEAT repository are:

- **CaseStudy**: the table contains the relevant metadata about a user case study related to the pathology under examination (i.e. case study identifier, name and description, BEAT session identifier, user id, etc.);
- **UserTable**: the table contains metadata about a registered user in the web interface;
- **HuEx_1_0_st_v2_probeset, HuEx_1_0_st_v2_transcript**: tables containing data extracted from the Affymetrix annotation files for Human Exon Array;
- **Hugo, HugoAliases:** tables containing human gene name and information extracted from the HGNC database;
- **aspic_huex, aspic_huex_location, Exon, Gene, Transcript, Transcript_Exon:** tables containing data extracted from ASPicDB, useful to represent exon signal on predicted transcripts;
- **pathway_genes, pathway_name:** tables containing metabolic pathways and gene names correlations extracted from KEGG, BioCyc and BioCarta Pathways databases.

## Data marts

BEAT data marts are designed to represent genetic multidimensional data with the aim of structuring bioinformatics data aggregations at both exon and gene level. They are focused on the organization of the data produced, in order to optimize the queries executed by the web front-end. The BEAT data marts were designed using the fact constellation schema conceptual model and adopting the standard Dimensional Fact Model graphical annotation.

The two data marts developed to support exon and gene level statistical analyses, are defined with different fact tables and hierarchies, due to the different conceptual organization of data (focused respectively on exon/probeset signals and transcript cluster/gene signals). Table 1 and Table 2 list the fact tables contained in both data marts reporting details on facts and measures. Table 3 lists the hierarchies and their attributes. Conceptual design phase has led to the definition of the same dimensional tables for both data marts. They are originated from the clinical information provided as input by the user and they are designed for the multidimensional analyses of expression data. Table 4 reports the definition and details of the dimensional tables.

The dimensional, hierarchical, and fact tables are created and populated by an ETL process that uses a set of data sources representing the input data-flow of the process. The data source set was composed by data extracted from the repository and from the semi-structured files containing the results of the exon and gene level statistical and meta statistical analyses performed.

# Tables

## Table 1 - Fact tables implemented in BEAT_exp_exonlevel

| **Fact Table** | **Facts** | **Measures** |
| --- | --- | --- |
| fact_exon | probeset_id  transcrip_cluster_id  Experiment | Exon array probeset Signal |
| fact_exon_expr_norm | probeset_id  transcript_cluster_id  Experiment  Gender  Age  Grading  Stage | probeset signal normalized by transcript cluster signal |
| fact_exon_expr_stats | id_fact_exon_expr_stats  probeset_id  transcript_cluster_id | Fold Change,  Splicing Index,  p-value (t-test) |
| fact_exon_expr_dabg, | transcrip_cluster_id  probeset_id  Experiment | p-value (DABG) |
| fact_exon_groups | probeset_id | output of the decision tree for multidimensional analysis |
| fact_exon_midas | probeset_idtranscrip_cluster_id | p-value (MIDAS) |

## Table 2 - Fact tables implemented in BEAT_exp_genelevel

| **Fact table** | **Facts** | **Measures** |
| --- | --- | --- |
| fact_gene | ID_FACT_GENE,  transcript_cluster_id,  Experiment | Exon array transcript_cluster Signal |
| fact_gene_tcid_expr | id_fact_gene_tcid_expr  transcript_cluster_id  GeneName  Experiment | Exon array transcript_cluster Signal |
| fact_gene_tcid_meta | id_fact_gene_tcid_meta  GeneName  transcript_cluster_id | Max absolute Splicing index (SI),  SI coefficient of variation,  Min pvalue (t-test),  Mean pvalue (MIDAS),  Min pvalue (MIDAS),  probeset_count,  Alternative Splice event count |
| fact_gene_tcid_stats | id_fact_gene_tcid_stats  transcript_cluster_id  GeneName | Fold Change  pvalue (t-test) |

## Table 3 - Hierarchies implemented in BEAT_exp_genelevel data mart

| **Hierarchies** | **Description** |
| --- | --- |
| agg_geneassignment_HugoAliases | Denormailzed table to correlate the official (HGNC) gene names and aliases to the Affymetrix annotation gene names |
| agg_mrnageneassignment_HugoAliases | Denormalized table to correlate transcript cluster_id and gene names (Affymetrix annotation files), gene names and aliases (HGNC) |
| agg_gene_tcid_GO_Pathway | Denormalized table to correlate gene names (HGNC), transcript cluster_id (Affymetrix annotation file), Gene Ontology terms and Pathway names |
| agg_Aspic_Gene_Transcript_Exon | Denormalized table to correlate exon, transcript and gene annotation for predicted transcript (ASPicDB) |

## Table 4 - Dimensional tables definition

| **Dimensions** | **Description** |
| --- | --- |
| Dim_experiment | reports experiments metadata |
| Dim_Gender | reports the patient gender |
| Dim_TissueType | reports the cancer tissue |
| Dim_Ageonset | reports the patients age of cancer onset |
| Dim_Grading | reports the tumor grade classification |
| Dim_Stage | reports the extent or severity of the cancer |
